# Supplementary figures and images for: Biomarker selection depends on gene function and organ: the case of the cytochrome P450 family genes in freshwater fish exposed to chronic pollution
Source: PeerJ. 2024 Feb 14;12:e16925. doi: 10.7717/peerj.16925 (PMC10874176; doi:10.7717/peerj.16925)

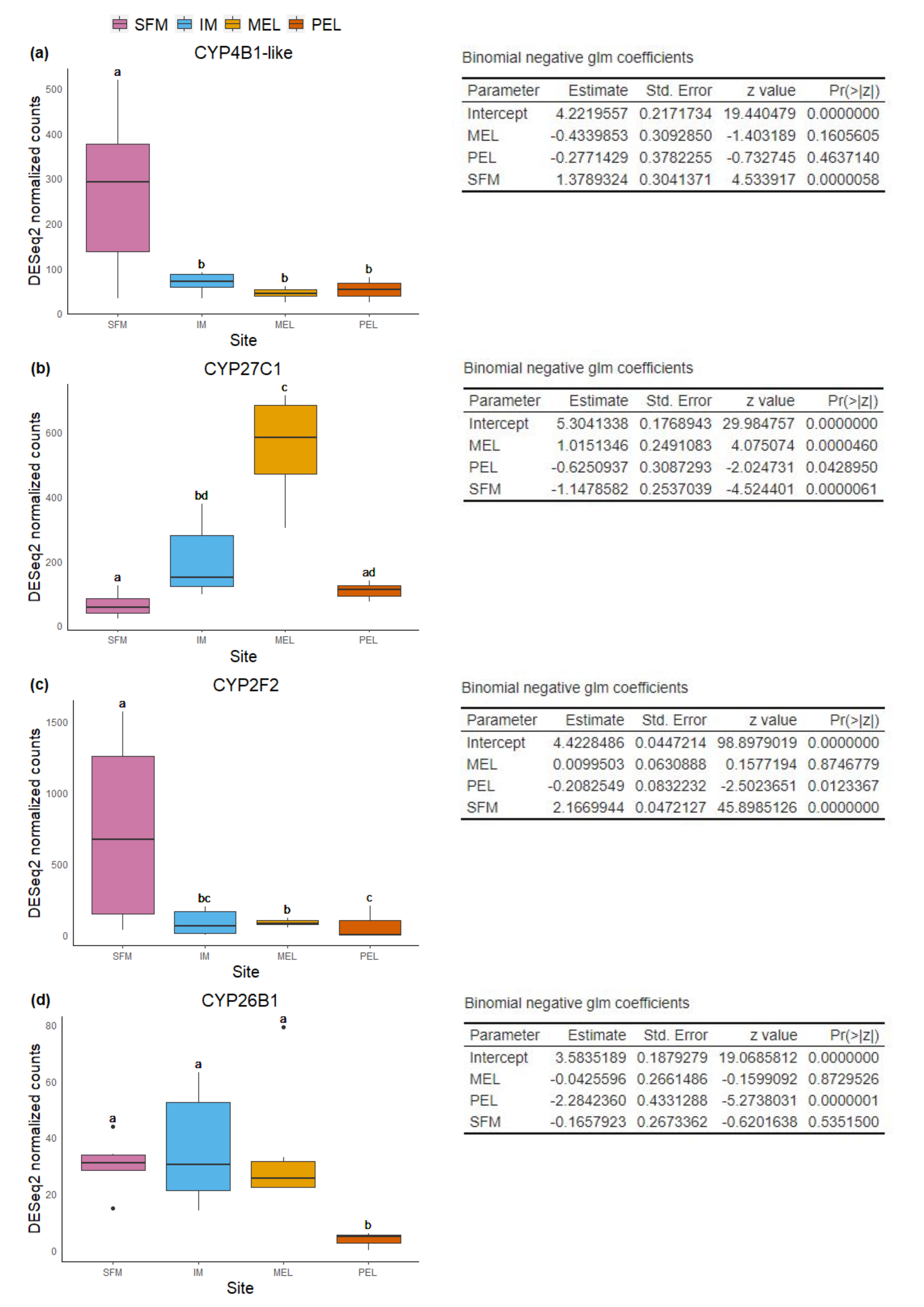

Supplement: Supplemental Information 1 — Boxplot of normalized read counts and model coefficients for CYP4B1-like (a), CYP27C1 (b), CYP2F2 (c), and CYP26B1 (d) in liver. [file peerj-12-16925-s001.png]

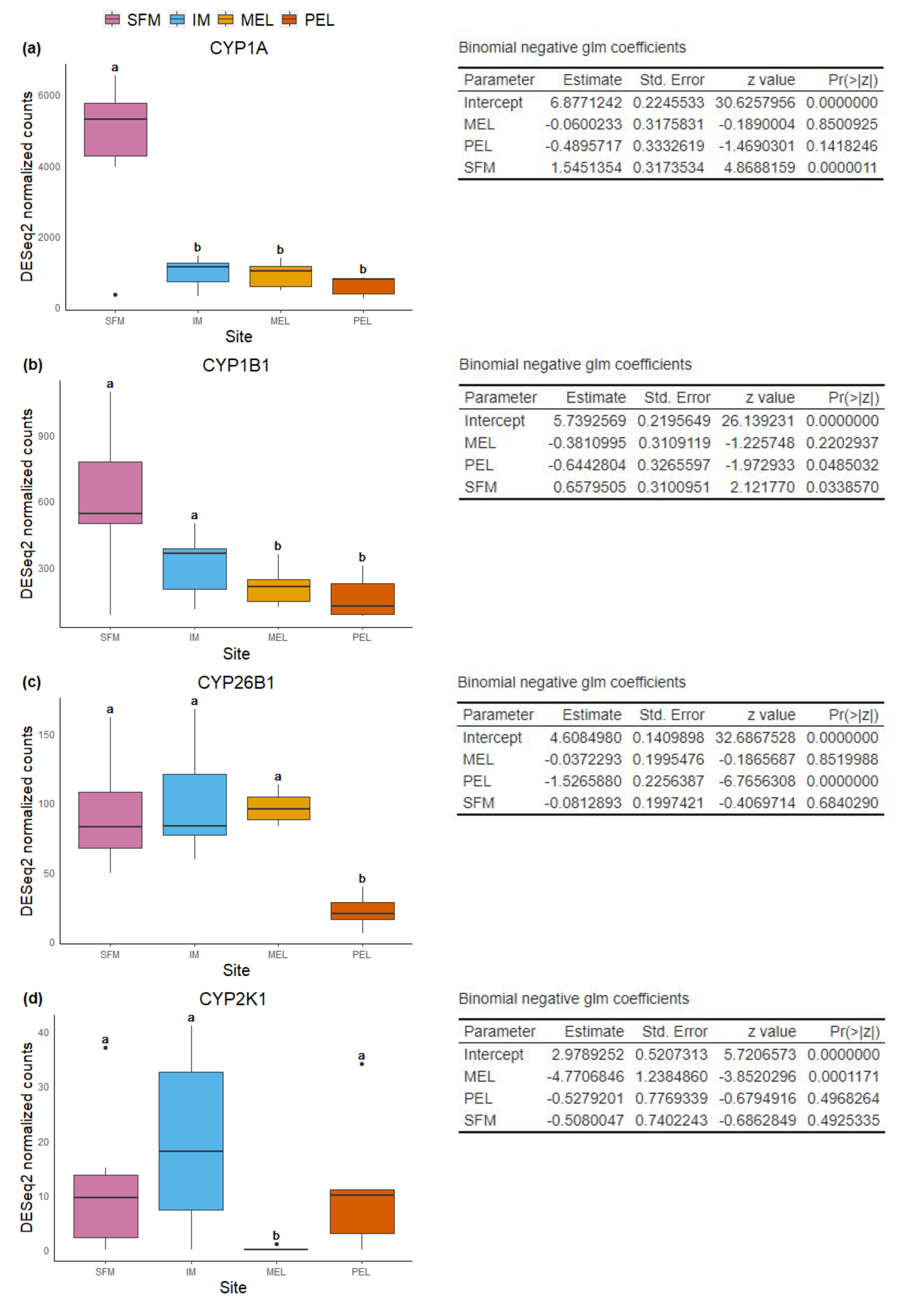

Supplement: Supplemental Information 2 — Boxplot of normalized read counts and model coefficients for CYP1A (a), CYP1B1 (b), CYP26B1 (c), and CYP2K1 (d) in gill. [file peerj-12-16925-s002.png]
